# Degenerative Disease of Intervertebral Disc: A Narrative Review of Pathogenesis, Clinical Implications and Therapies

**DOI:** 10.3390/bioengineering13010040

**Published:** 2025-12-29

**Authors:** Lidija Gradisnik, Nina Kocivnik, Uros Maver, Tomaz Velnar

**Affiliations:** 1 Institute of Biomedical Sciences, Medical Faculty of Maribor, 2000 Maribor, Slovenia; lidija.gradisnik@um.si (L.G.);; 2 Faculty of Pharmacy, University of Ljubljana, 1000 Ljubljana, Slovenia; 3 Department of Neurosurgery, University Medical Centre Ljubljana, 1000 Ljubljana, Slovenia; 4 Faculty of Health Sciences, Alma Mater Europaea University, 2000 Maribor, Slovenia

**Keywords:** intervertebral disc, degenerative disc disease, oxidative stress, cytokines, cellular senescence

## Abstract

This narrative review examines degenerative disc disease (DDD), a major cause of chronic back pain and disability worldwide. It is a multifactorial condition resulting from a complex interplay of genetic, mechanical, metabolic, and environmental factors that progressively impair disc structure and function. The pathophysiology of DDD involves disruption of extracellular matrix homeostasis, cellular senescence, oxidative stress, and chronic inflammation mediated by cytokines such as IL-1β, TNF-α, and IL-6. These processes are further modulated by signalling pathways including NF-κB, MAPK, and Wnt/β-catenin, leading to matrix degradation, dehydration, and loss of disc height. Epidemiological studies highlight the contribution of lifestyle and metabolic disorders, such as obesity, smoking, and diabetes, to disease progression. Traditional conservative and surgical treatments primarily alleviate symptoms but do not halt or reverse degeneration. In contrast, recent advances in molecular biology and regenerative medicine have opened new therapeutic avenues. Mesenchymal stem cell therapy, biomaterial scaffolds, and gene-based interventions aim to restore disc homeostasis by promoting matrix synthesis and suppressing catabolic activity. Despite promising experimental results, clinical translation remains limited by challenges in cell viability, delivery methods, and long-term efficacy. Future research integrating molecular, biomechanical, and regenerative strategies offers the potential for true biological repair and disc regeneration.

## 1. Introduction

Degenerative disc disease (DDD) and its associated back pain represent a major and increasing global health challenge [1]. These conditions are chronic, multifactorial, and contribute significantly to both morbidity and disability across populations. Although the term “degeneration” suggests a simple wear-and-tear mechanism, the pathophysiology of DDD is far more complex. It involves a dynamic interplay of mechanical forces, genetic predisposition, metabolic alterations, nutritional constraints, and environmental influences. Together, these factors progressively impair the structure and function of the intervertebral disc (IVD), ultimately compromising the biomechanical integrity of the spinal segment [2,3].

The IVD was long regarded merely as a passive “shock absorber” between adjacent vertebral bodies, but it is now recognised as a metabolically active tissue that maintains homeostasis through cellular and extracellular matrix (ECM) processes [3,4,5]. When this homeostasis is disrupted, degenerative changes occur, which may manifest clinically in various ways. Patients with DDD may present with axial back pain, localised to the spinal column itself, as well as more complex syndromes such as spinal stenosis (narrowing of the spinal canal), myelopathy (spinal cord dysfunction, in this case due to the IVD compression), or radiculopathy (nerve-root compression). These clinical presentations are not only symptomatic burdens, but also reflect underlying structural compromise and may contribute to chronic spinal instability and progressive functional impairment. The impact is not restricted to older adults; younger and working-age individuals may also experience significant reductions in quality of life, productivity, and activity levels [1,2].

Despite decades of research, many critical questions remain regarding the earliest molecular and cellular events that drive IVD degeneration, the transition from structural IVD damage to symptomatic pain, and the most effective strategies to arrest or reverse the process [4,5]. Historically, interventions have focused on symptom relief, through analgesia, physical therapy, and surgery. However, this often allows underlying degeneration to continue unabated. Recent advances in molecular diagnostics, imaging modalities, and regenerative medicine are beginning to elucidate the biochemical and cellular mechanisms underlying DDD, offering new opportunities for targeted and biologically oriented treatments [4,5,6,7]. This narrative review aims to summarise the mechanisms, risk factors, current treatments, and emerging regenerative approaches for DDD, and to outline key challenges and future therapeutic directions.

## 2. Anatomy of the Intervertebral Disc

**Figure 1:**
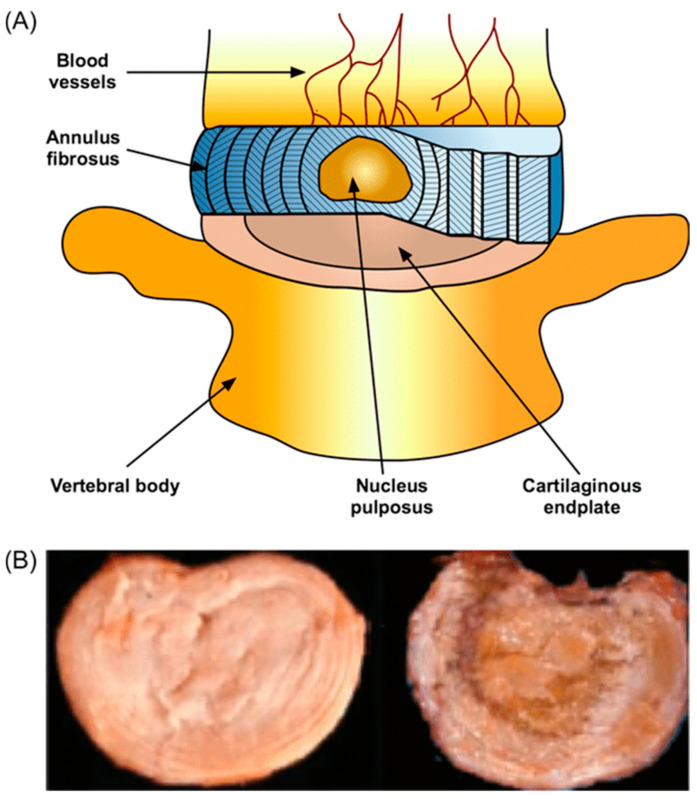
A schematic representation of IVD anatomy (**A**) and two images of healthy (**left**) and degenerated IVD (**right**) (**B**) (with permission from [12]).

To understand the degenerative processes, it is essential to briefly review the IVD’s normal anatomy and physiology (Table 1). The IVD comprises three major structural components: the outer annulus fibrosus (AF), the central nucleus pulposus (NP), and the superior and inferior cartilaginous endplates (CEPs) (Figure 1). The AF consists of concentric lamellae of collagen (primarily type I in the outer AF and type II in the inner AF), which resist tensile and shear forces [8,9,10]. The NP is a hydrated, proteoglycan-rich gel within the AF, able to respond to compressive loads by developing hydrostatic pressure, largely due to the high concentration of aggrecan and associated glycosaminoglycan (GAG) chains. The CEPs serve as the interface between the IVD and adjacent vertebral bodies and permit diffusion of nutrients and metabolites to IVD cells. The IVD is avascular, with no direct blood vessels within the NP and inner AF; instead, nutrient exchange occurs via diffusion across the CEPs from small capillaries in adjacent vertebral subchondral bone [6,7,8,11].

**Table 1:** The summary of normal anatomy and physiology of IVD.

Component/ Feature	Structure/Composition	Function	Key Physiological Characteristics	References
**Annulus** **fibrosus (AF)**	Concentric collagen lamellae Outer AF: mainly Type I collagen Inner AF: mainly Type II collagen	Resists tensile and shear forces Maintains structural containment of NP	Limited vascularity (inner AF avascular) Provides circumferential strength	[4,13,14]
**Nucleus** **pulposus (NP)**	Hydrated, gel-like core Rich in proteoglycans, especially aggrecan High GAG content enabling water retention	Absorbs and redistributes compressive loads via hydrostatic pressure	Completely avascular Maintains hydration and pressure-dependent load distribution	[6,8,15]
**Cartilaginous endplates (CEPs)**	Superior and inferior hyaline cartilage layers Interface between IVD and vertebral bodies	Allow diffusion of nutrients, oxygen, and metabolites to IVD cells	Primary route for nutrient/waste exchange Dependent on capillaries in subchondral bone	[16,17,18]
**Vascular** **supply**	NP and inner AF: no direct blood vessels CEPs adjacent to vertebral capillary network	Enables diffusion-based nutrient delivery and waste removal	Slow metabolic turnover Vulnerability to hypoxia and nutrient limitations	[17,19,20]
**Cellular** **activity**	Sparse disc cells regulating ECM Balance between anabolic and catabolic activity	Maintains ECM structure and disc integrity	Low metabolic reserve Sensitive to nutrient deficits and mechanical stress	[16,21,22,23]
**Biomechanical function**	Coordinated function of AF, NP, CEPs	Smooths distribution of compressive, tensile, and shear forces	Homeostasis depends on hydration, matrix turnover, and cell viability	[1,4,16]
**Physiological vulnerabilities**	Avascular structure Hypoxic microenvironment Limited nutrient supply and waste removal	Predispose disc to degeneration when homeostasis is disrupted	Increased risk under mechanical load and with aging	[14,17,24]

Under normal physiological loading, the IVD distributes compressive, tensile, and shear forces smoothly through its structure, maintaining hydration, matrix synthesis, and cellular viability. IVD cells, though sparse, actively regulate the ECM, balancing anabolic and catabolic processes. Proteoglycans sequester water, collagen fibres impart structural strength, and cells produce and degrade ECM in a dynamic flux. However, this homeostatic balance is vulnerable. Because of the IVD’s avascular nature, limited nutrient delivery, waste removal, and hypoxic environment, it has relatively low metabolic reserve. Combined with mechanical burden and age-related changes, this predisposes the IVD to degeneration [9,13,14,16].

## 3. The Multifactorial Nature of Intervertebral Disc Degeneration

IVD degeneration is not caused by a single factor but is the result of multiple intersecting pathways. Although mechanical stress may be a major initiating factor in DDD, it does not act independently. The degenerative process arises from a multifactorial interplay involving biochemical imbalances, inflammatory mediators, genetic predispositions, and age-related cellular changes, all of which compromise the IVD’s capacity for repair and maintenance [14,16,21]. Elucidating these interdependent mechanisms is crucial for a comprehensive understanding of the pathophysiology of IVD degeneration and for developing targeted therapeutic strategies [22,23,25]. The multifactorial nature of the discussed IVD degeneration is summarised in Table 2.

**Table 2:** Multifactorial mechanisms of IVD degeneration.

Category	Representative Factors	Key Mechanisms	Consequences for IVD	References
**Mechanical stress and load**	Heavy labour, torsion, bending, vibration exposure	Repetitive microtrauma to AF and NP Collagen/elastin disorganisation Loss of NP hydration Altered biomechanics → increased shear forces	Decreased disc height Structural failure Instability Herniation and nerve compression	[1,8,16,26]
**Genetic and environmental factors**	Gene variants (*COL1A1, COL9A2, ACAN, IL-1, IL-6*), smoking, obesity, vibration, repetitive loading	SNPs affecting ECM proteins and inflammatory mediators Altered ECM synthesis or stability Epigenetic changes due to lifestyle factors	Early weakening of ECM Increased inflammatory signalling Catabolic microenvironment	[27,28,29,30,31]
**Nutritional and metabolic aspects**	Endplate calcification, atherosclerosis, diabetes, metabolic syndrome, obesity	Impaired nutrient diffusion across CEP Local hypoxia + acidic microenvironment Accumulation of AGEs Oxidative stress and systemic metabolic dysregulation	Reduced cell viability Inhibited proteoglycan synthesis ECM stiffening Impaired permeability and degeneration	[17,19,32,33,34]
**Cellular senescence**	Increased ROS, mitochondrial dysfunction, SASP factors (IL-1β, IL-6, TNF-α), MMPs, ADAMTS	Oxidative stress, DNA damage, mitochondrial dysfunction SASP production: pro-inflammatory cytokines, MMPs, ADAMTS NF-κB and p38 MAPK activation	ECM degradation Increased inflammation Loss of regenerative capacity Accumulation of non-functional cells	[35,36,37,38,39,40]
**Ageing and microenvironmental changes**	Age-related CEP thickening, decreased metabolic activity, reduced nutrient diffusion	Loss of proteoglycans and GAGs → decreased hydration Increased collagen cross-linking, fragmentation, CEP calcification and sclerosis Accumulation of waste products	Reduced elasticity and load-distribution Hypoxia and acidity Increased apoptosis and catabolism Progressive irreversible degeneration	[14,17,20,41]
**Lifestyle and comorbidities**	Smoking, obesity, poor posture, inactivity, diabetes	Smoking-induced hypoxia Obesity increasing axial load Sedentary behaviour reducing beneficial mechanical stimuli Metabolic disorders increasing AGEs	Oxidative stress Matrix degradation Increased stiffness and reduced tensile strength Accelerated degeneration	[20,24,32,42,43]
**Hormonal changes**	Decline in estrogen levels (e.g., menopause) Altered androgen (testosterone) levels Reduced growth hormone (GH)/insulin-like growth factor-1 (IGF-1) signalling Increased cortisol (glucocorticoids) due to chronic stress or aging Dysregulation of thyroid hormones Altered parathyroid hormone (PTH) and vitamin D axis Hormone changes associated with aging, metabolic syndrome, and diabetes	Estrogen deficiency: ↓ proteoglycan and collagen synthesis ↑ inflammatory cytokines (IL-1β, TNF-α) ↑ oxidative stress and apoptosis in NP cells Reduced IGF-1/GH signalling: ↓ anabolic ECM turnover ↓ disc cell proliferation and survival impaired matrix repair capacity Elevated cortisol: Suppression of ECM synthesis promotion of catabolic and apoptotic pathways increased sensitivity to mechanical stress Thyroid hormone imbalance: Altered cellular metabolism and matrix homeostasis Disrupted disc cell differentiation Vitamin D deficiency/altered PTH Impaired endplate metabolism Reduced nutrient diffusion Increased calcification risk Hormone–inflammation crosstalk Amplification of catabolic microenvironment Enhanced NF-κB and MAPK signalling	Reduced proteoglycan and water content Accelerated nucleus pulposus dehydration Impaired extracellular matrix maintenance Increased disc cell senescence and apoptosis Reduced regenerative potential Enhanced susceptibility to mechanical overload Faster progression of age- and sex-related disc degeneration Increased prevalence of degeneration in post-menopausal individuals	[44,45,46,47,48,49,50]

### 3.1. Mechanical Stress and Load

Abnormal or sustained mechanical loading, such as that experienced during heavy physical labour, repetitive bending, torsion, or exposure to vibration, may impose continuous microtrauma on the IVD components [3,4,5]. These repetitive mechanical stresses disrupt the structural integrity of the AF, leading to the formation of microfissures and disorganisation of collagen and elastin fibres within the ECM. Consequently, the NP progressively loses its gel-like consistency and water-binding capacity, diminishing its ability to distribute compressive loads evenly across the IVD. The resulting reduction in IVD height alters the normal biomechanics of the spinal motion segment, increasing shear forces and abnormal stress on adjacent vertebrae and facet joints [6,7]. Over time, these maladaptive changes propagate a cycle of mechanical instability and cellular stress that accelerates degenerative processes in neighbouring IVDs. Ultimately, this can culminate in structural failure, IVD herniation, or nerve root compression, manifesting clinically as pain, restricted mobility, or neurological symptoms [26,51,52].

### 3.2. Genetic and Environmental Factors

Genetic predisposition plays a significant role in the development of DDD. Numerous studies have identified single-nucleotide polymorphisms (SNPs) in genes encoding key structural and regulatory proteins of the IVD, including collagens (*COL1A1*, *COL9A2*), aggrecan (*ACAN*), and interleukins (*IL-1, IL-6*), as important determinants of individual susceptibility [27,28]. Variants in these genes can alter the synthesis, composition, or stability of the ECM and influence inflammatory signalling pathways, thereby predisposing the IVD to early structural weakening and catabolic imbalance. However, genetic background alone does not fully explain the variability in disease onset or progression. Environmental and lifestyle factors, such as repetitive mechanical loading, vibration exposure, smoking, obesity, and metabolic disorders, play critical modulatory roles. These factors can induce or amplify pathogenic processes by influencing gene expression through epigenetic modifications, including DNA methylation, histone acetylation, and the activity of non-coding RNAs [27,28,29,52,53]. For instance, cigarette smoke and chronic mechanical stress have been shown to alter the methylation status of matrix-degrading enzymes and inflammatory mediators, thereby promoting a catabolic microenvironment within the IVD [15,35,54].

This complex interplay between genetic susceptibility and environmental exposure reinforces the concept of DDD as a multifactorial disorder, shaped by both inherited and acquired influences. Understanding these interactions is crucial for identifying at-risk individuals and developing personalised preventive and therapeutic strategies targeting not only structural integrity but also the underlying molecular and epigenetic mechanisms of IVD degeneration [29,52].

### 3.3. Nutritional and Metabolic Aspects

Due to its largely avascular structure, the IVD depends on diffusion through the adjacent CEP to obtain nutrients and oxygen necessary for cellular survival and matrix maintenance [55,56]. This diffusion process is tightly regulated and highly sensitive to alterations in endplate permeability and vascular supply. When compromised, through factors such as endplate calcification, microvascular atherosclerosis, or systemic hypoxia, nutrient exchange becomes inefficient. As a result, metabolites such as lactic acid accumulate, lowering the local pH and creating an acidic microenvironment that suppresses cell viability, inhibits proteoglycan synthesis, and accelerates ECM degradation [17,55,56,57].

Beyond local vascular and oxygenation factors, systemic metabolic dysregulation has emerged as an important contributor to IVD degeneration. Impaired glucose metabolism and insulin resistance, commonly observed in metabolic syndrome and diabetes, promote oxidative stress and increase the formation of advanced glycation end-products (AGEs) [17,32,57]. These AGEs form irreversible cross-links within collagen fibres, reducing their elasticity and altering the biomechanical and transport properties of the IVD matrix. The resulting tissue stiffening and impaired permeability further limit nutrient diffusion, establishing a vicious cycle of metabolic and structural deterioration [33,34].

Moreover, obesity and related metabolic disorders can exacerbate these effects through chronic low-grade inflammation and increased production of pro-inflammatory cytokines and reactive oxygen species (ROS), both of which accelerate IVD cell senescence and apoptosis. Collectively, these findings underscore that IVD degeneration is not solely a local, mechanically driven process but also a systemic metabolic disorder influenced by vascular health, nutritional supply, and cellular energy metabolism [36,58].

### 3.4. Cellular Senescence

Cellular senescence represents an evolutionarily conserved response to a range of intrinsic and extrinsic stressors, leading to an irreversible halt in cell proliferation. In the IVD, this process is central to both aging and degeneration, as it reduces the population of functional cells and alters the surrounding microenvironment [37,38].

Cellular senescence, oxidative stress, and inflammation are increasingly recognised as central molecular mechanisms in IVD degeneration. Accumulating evidence suggests that oxidative damage, mitochondrial dysfunction, and persistent DNA damage response activation trigger premature cellular senescence in IVD cells, particularly NP and AF cells [59]. Senescence represents a state of permanent cell cycle arrest accompanied by distinct phenotypic changes, including altered gene expression and metabolic activity. Senescent cells develop a senescence-associated secretory phenotype, characterised by the release of pro-inflammatory cytokines (IL-1β, IL-6, and TNF-α), chemokines, growth factors, and matrix-degrading enzymes such as matrix metalloproteinases (MMPs) and aggrecanases (a disintegrin and metalloproteinase with thrombospondin motifs—ADAMTS) [19,38,59,60]. These enzymes break down proteoglycans, mainly aggrecan, thus accelerating ECM loss and IVD degeneration. This secretory profile disrupts the local microenvironment, promoting ECM degradation, impairing matrix synthesis, and amplifying inflammation within the IVD. Over time, these changes establish a self-perpetuating cycle of catabolism and inflammation that accelerates tissue degeneration [19,60].

Furthermore, oxidative stress, resulting from increased ROS production and reduced antioxidant defences, further enhances senescence by inducing mitochondrial damage and DNA lesions. Mitochondrial dysfunction in IVD cells not only compromises ATP production but also increases ROS generation, amplifying the senescence signal [38,59]. The accumulation of senescent cells within the IVD contributes to a decline in regenerative capacity, as these cells exhibit impaired proliferative and anabolic potential [36,39,61].

Recent studies also suggest that the nuclear factor kappa B (*NF-κB*) and p38 mitogen-activated protein kinases (*p38 MAPK*) signalling pathways play crucial roles in regulating senescence-associated secretory phenotype (SASP) expression and sustaining the pro-inflammatory milieu in degenerated IVDs. Targeting these pathways, as well as developing strategies to eliminate senescent cells (senolytics) or suppress SASP (senomorphics), has emerged as a promising therapeutic avenue for slowing or reversing IVD degeneration [39,62,63].

### 3.5. Ageing and Microenvironmental Changes

Ageing is one of the most significant intrinsic factors contributing to IVD degeneration, as it profoundly alters the IVD’s cellular composition and ECM organisation [64,65]. With advancing age, the water-binding capacity of the NP diminishes due to a marked reduction in proteoglycan and glycosaminoglycan content, leading to dehydration and loss of IVD turgor. Concurrently, collagen fibres within both AF and NP undergo structural reorganisation, characterised by increased cross-linking, fragmentation, and disorientation, which compromise the IVD’s elasticity and mechanical resilience [36,37,58]. The CEPs, which play a crucial role in nutrient diffusion and metabolic exchange, progressively thicken, calcify, or become sclerotic, further restricting the transport of oxygen and nutrients to the avascular IVD cells. As a result, cellular metabolism declines, waste products accumulate, and the microenvironment becomes increasingly hypoxic and acidic. These microenvironmental shifts not only impair cellular viability and matrix synthesis but also promote catabolic processes and apoptosis. Functionally, the IVD’s reduced capacity to absorb compressive load increases its stiffness and predisposes it to fissuring, annular tearing, and bulging under normal physiological stress. Although signs of IVD degeneration may appear as early as adolescence, the cumulative effects of age-related molecular and structural changes lead to progressive and irreversible deterioration over time [37,38,54,59,65].

### 3.6. Lifestyle and Comorbidity Influences

A growing body of epidemiological and clinical evidence indicates that lifestyle factors such as smoking, obesity, sedentary behaviour, poor posture, and metabolic disorders significantly influence both the onset and progression of IVD degeneration [2,4,6]. Cigarette smoking, for example, induces vasoconstriction and reduces oxygen transport, thereby compromising the already limited nutrient supply to the avascular IVD tissue [30]. Additionally, the accumulation of nicotine and other toxins impairs IVD cell metabolism and increases oxidative stress, accelerating matrix degradation. Obesity increases axial loading on the spine, amplifying mechanical stress on the IVDs, and contributes to a chronic low-grade systemic inflammatory state that further disrupts IVD homeostasis. Similarly, a sedentary lifestyle and poor posture reduce spinal flexibility and diminish the beneficial mechanical stimuli necessary for maintaining healthy IVD metabolism [32,33,65,66]. Metabolic disorders such as diabetes mellitus exacerbate these effects by promoting the accumulation of advanced glycation end-products in collagen fibres, resulting in increased stiffness and reduced tensile strength of the ECM. Collectively, these risk factors interact through mechanical, biochemical, and inflammatory pathways, creating a hostile IVD microenvironment that accelerates degenerative changes and impairs regenerative potential [28,55,56].

### 3.7. Hormonal Influences on the Intervertebral Disc

Hormonal regulation plays a key modulatory role in maintaining IVD homeostasis, and increasing evidence suggests that age- and disease-related hormonal alterations contribute significantly to the onset and progression of DDD. Although the IVD is not a classical endocrine target tissue, IVD cells express receptors for several systemic hormones, indicating that endocrine signalling influences cellular metabolism, extracellular matrix (ECM) turnover, inflammation, and cell survival [6,7,13]. Hormonal changes therefore act as systemic modifiers that interact with mechanical, metabolic, and inflammatory stressors to shape the degenerative microenvironment of the IVD.

Among the most extensively studied hormonal influences is oestrogen. Oestrogen receptors (ERα and ERβ) have been identified in NP and AF cells, supporting a direct regulatory role of oestrogen in IVD biology [6,7,44]. Oestrogen promotes proteoglycan and collagen synthesis, enhances IVD cell viability, and suppresses inflammatory cytokine production. Consequently, oestrogen deficiency, such as that occurring during menopause, is associated with accelerated DDD, reduced IVD height, and increased prevalence of degenerative changes on imaging [6,24]. Experimental studies demonstrate that oestrogen withdrawal results in decreased aggrecan synthesis, increased matrix metalloproteinase (MMP) activity, elevated oxidative stress, and enhanced apoptosis of NP cells, all of which contribute to IVD dehydration and structural failure [13,14,38,44,45]. Clinically, postmenopausal women show a higher incidence and severity of IVD degeneration, supporting the concept that oestrogen exerts a protective effect on IVD tissue [6,24]. In addition, oestrogen deficiency may adversely affect vertebral endplate metabolism, further impairing nutrient diffusion to the avascular IVD and exacerbating degeneration [17,41,45,46,47].

Androgens, including testosterone, may also influence IVD health, although their role is less well defined. Androgen receptors have been detected in IVD cells, and androgens are thought to support anabolic metabolism and cellular survival [7,9]. Age-related declines in testosterone levels, particularly in men, may therefore contribute to reduced ECM synthesis and diminished regenerative capacity of the IVD. While direct mechanistic data remain limited, epidemiological observations indicate sex-related differences in DDD prevalence and progression that may partially reflect different hormonal environments [6,24,44].

Growth hormone (GH) and its downstream mediator insulin-like growth factor-1 (IGF-1) are central regulators of tissue growth, metabolism, and repair. IGF-1 is a potent anabolic factor for IVD cells, stimulating proteoglycan and collagen synthesis, enhancing cell proliferation, and inhibiting apoptosis [7,21,48]. IVD cells express IGF-1 receptors, and reduced IGF-1 signalling has been associated with impaired matrix maintenance and increased susceptibility to degeneration [13,16]. Age-related decline in GH/IGF-1 axis activity may therefore contribute to the progressive loss of IVD cellularity and ECM integrity observed during ageing. Conversely, dysregulated GH signalling has also been linked to altered IVD metabolism, underscoring the need for balanced endocrine regulation [7,22,48].

Glucocorticoids, particularly cortisol, represent another important hormonal influence on DDD. Chronic elevation of glucocorticoids, as observed in prolonged stress states or long-term steroid exposure, exerts predominantly catabolic effects on musculoskeletal tissues. In the IVD, glucocorticoids suppress proteoglycan synthesis, reduce cell proliferation, and promote apoptosis and cellular senescence [37,38,49]. They also enhance oxidative stress and may increase the expression of inflammatory mediators and matrix-degrading enzymes, thereby accelerating degenerative processes. Although short-term glucocorticoid exposure may exert anti-inflammatory effects, chronic exposure is likely detrimental to IVD homeostasis [14,60].

Thyroid hormones regulate cellular metabolism, differentiation, and energy homeostasis and may indirectly affect IVD biology. Alterations in thyroid hormone levels have been associated with changes in cartilage metabolism and bone-cartilage interactions, suggesting potential effects on IVD cells and vertebral endplates [20,57]. Thyroid hormone imbalance may influence cell metabolic activity, ECM turnover, and responsiveness to mechanical loading, although direct mechanistic evidence remains limited and warrants further investigation [50].

The vitamin D–parathyroid hormone (PTH) axis constitutes an additional endocrine pathway relevant to DDD. Vitamin D deficiency is common in ageing and metabolically compromised populations and has been associated with musculoskeletal degeneration. Vitamin D is involved in calcium homeostasis, modulation of inflammation, and cellular differentiation. In the context of IVDD, altered vitamin D and PTH signalling may contribute to endplate calcification, impaired nutrient diffusion, and reduced IVD cell viability, thereby indirectly promoting degeneration [17,20,32].

Importantly, hormonal changes rarely occur in isolation. Instead, they interact with mechanical overload, inflammation, oxidative stress, and metabolic dysregulation to amplify degenerative pathways. Hormonal deficiency or imbalance can lower the threshold for IVD injury, reduce repair capacity, and accelerate cellular senescence. These interactions highlight the systemic nature of DDD and underscore the importance of considering endocrine status when evaluating disease risk and progression. Collectively, hormonal alterations represent a significant, yet under-recognised, component of the multifactorial pathophysiology of DDD [36,37,38].

## 4. Molecular and Cellular Mechanisms of Degeneration

Biochemical and molecular studies have revealed a complex cascade of cellular and extracellular events underlying IVD degeneration. Rather than being a passive process of tissue wear, IVD degeneration is an active, multifactorial interplay between mechanical stress, inflammation, oxidative damage, and altered cell–matrix signalling [58].

ECM breakdown and loss of proteoglycans are among the main features of IVD degeneration, resulting from cell senescence. During degeneration, progressive proteoglycan loss and fragmentation reduce osmotic pressure, leading to dehydration, IVD shrinkage, and impaired mechanical function [2,3,37,38]. Breakdown products may diffuse out of the IVD, while the collagen network becomes disorganised, further compromising biomechanical integrity. Increased catabolic enzyme activity is a hallmark of degeneration, with upregulation of matrix-degrading enzymes, including MMPs, aggrecanases of the ADAMTS family, and cathepsins. These enzymes cleave collagen and aggrecan, disrupting ECM architecture and generating degradation products that amplify inflammatory signalling and cellular stress [38,58].

IVD cells, as well as adjacent vertebral endplate and immune cells, secrete and respond to pro-inflammatory cytokines. These cytokines enhance the expression of catabolic enzymes, suppress anabolic matrix synthesis, and promote apoptosis or senescence of IVD cells. Chronic cytokine exposure also facilitates nerve and vessel ingrowth into previously immune-privileged regions of the IVD, contributing to pain generation and neuroinflammation. Increasing evidence indicates that these cytokines act downstream of more complex molecular regulatory networks, rather than serving as primary drivers of degeneration. Recent molecular discoveries have expanded this concept, identifying epigenetic regulation, post-transcriptional control by non-coding RNAs, and cellular senescence as central drivers of DDD [19,38,67].

Cellular senescence has emerged as a key molecular mechanism linking ageing, inflammation, and matrix degradation in IVD degeneration. Senescent NP and AF cells accumulate with age and degeneration, and are characterised by irreversible cell-cycle arrest and the development of a SASP. The SASP involves sustained secretion of pro-inflammatory cytokines, chemokines, growth factors, and matrix-degrading enzymes such as IL-1β, IL-6, TNF-α, MMPs, and ADAMTS proteases [38,39]. Notably, SASP factors exert paracrine effects, inducing senescence in neighbouring cells and maintaining a chronic inflammatory and catabolic microenvironment. Activation of *NF-κB*, *p38 MAPK*, and *JAK/STAT* signalling pathways plays a pivotal role in sustaining SASP expression and amplifying tissue degeneration [39,62,63]. As senescent cells display impaired anabolic capacity but high catabolic activity, their accumulation leads to a progressive loss of regenerative potential within the IVD. The biomechanical alterations described above lead to structural failure. As the IVD loses hydration and height, its ability to evenly distribute mechanical loads across the spinal motion segment diminishes [2,3,4]. The healthy NP normally acts as a hydrostatic cushion, absorbing compressive forces and transmitting them uniformly to the AF and vertebral endplates. With degeneration, the loss of proteoglycans and water reduces the NP’s pressurisation capacity, shifting the load-bearing responsibility to the AF and posterior spinal elements. Additionally, the structural and metabolic integrity of the IVD depends heavily on the health of the adjacent vertebral CEPs. Degenerative changes such as endplate calcification, sclerosis, and microvascular insufficiency impair nutrient diffusion and waste removal. This results in reduced cellular viability, accumulation of metabolic byproducts, and further compromise of IVD homeostasis [17,21]. The altered load distribution results in abnormal mechanical stress on the annular lamellae, endplates, facet joints, and surrounding ligaments. The AF experiences increased tensile and shear forces, which promote collagen fibre disorganisation, annular fissures, and eventually radial tears. Similarly, the vertebral endplates may undergo microfractures and sclerosis, further impairing nutrient diffusion into the IVD and perpetuating the degenerative cycle [19,36].

To compensate for these biomechanical changes, adjacent spinal structures adapt, often in pathological ways. The facet joints are exposed to increased compressive loads, resulting in facet joint hypertrophy and osteophyte formation at vertebral margins. Simultaneously, the ligamentum flavum and other spinal ligaments may thicken in response to chronic mechanical stress, contributing to canal narrowing and spinal stenosis [2,3,4,63]. The paraspinal musculature may also become overactive or imbalanced, further exacerbating mechanical instability. In advanced stages, structural failure of the IVD, such as annular fissures or herniation of NP material, creates pathways for the ingrowth of nociceptive nerve fibres and blood vessels into normally avascular regions of the IVD. This neoinnervation, together with local production of pro-inflammatory mediators such as TNF-α and IL-1β, sensitises nerve endings and leads to discogenic pain, a major clinical manifestation of IVD degeneration [40,58].

Targeting senescent cells and their secretory phenotype has recently emerged as a promising therapeutic strategy for IVD degeneration. Senolytic agents selectively eliminate senescent cells by disrupting their reliance on anti-apoptotic pathways, while senomorphic agents suppress SASP activity without inducing cell death [62,63,64]. Preclinical studies have shown that clearance of senescent IVD cells or pharmacological inhibition of SASP signalling reduces inflammation, restores ECM synthesis, and attenuates IVD degeneration [63,64,65]. Compounds such as dasatinib, quercetin, and fisetin have demonstrated senolytic activity in musculoskeletal tissues, whereas inhibitors of *NF-κB*, *p38 MAPK*, and *JAK/STAT* pathways act as senomorphics by dampening SASP-driven inflammation [62,63,64]. These approaches represent a paradigm shift from symptom-based management towards targeting fundamental ageing-related mechanisms underlying IVD degeneration.

While inflammatory cytokines, catabolic enzymes, and biomechanical alterations are well-established downstream features of DDD, these processes alone do not fully account for the initiation and sustained progression of the degenerative cascade. Increasing evidence indicates that inflammatory and catabolic responses are governed by upstream regulatory mechanisms that integrate mechanical loading, metabolic stress, and inflammatory stimuli at the post-transcriptional and epigenetic levels. Persistent activation of cytokine signalling and matrix degradation pathways, even in the absence of acute inflammatory triggers, suggests the presence of molecular networks capable of maintaining pathological gene expression programmes within IVD cells [68]. In this context, non-coding RNAs have emerged as critical regulators of cell homeostasis, modulating inflammatory signalling, extracellular matrix turnover, oxidative stress responses, and cell fate decisions. Recognition of these upstream regulatory layers provides a more integrated framework for understanding IVD degeneration and supports a shift from purely downstream inflammatory mediators towards post-transcriptional control mechanisms as central drivers of disease progression [69].

### Post-Transcriptional Regulation by microRNAs and Long Non-Coding RNAs

Post-transcriptional regulation by non-coding RNAs has emerged as a critical molecular mechanism governing IVD cell homeostasis, inflammation, ECM turnover, and survival. Among these regulators, microRNAs (miRNAs) and long non-coding RNAs (lncRNAs) play central roles by fine-tuning gene expression in response to mechanical stress, inflammatory stimuli, oxidative damage, and metabolic perturbations. Increasing evidence indicates that dysregulation of these non-coding RNA networks is a key driver of DDD rather than a secondary epiphenomenon [68,69].

MicroRNAs are small, comprising up to 22-nucleotide non-coding RNAs that repress gene expression by binding to complementary sequences in target mRNAs, leading to translational inhibition or mRNA degradation. Multiple profiling studies have demonstrated distinct miRNA expression signatures in degenerated NP and AF tissues compared with healthy IVD. Pro-degenerative miRNAs such as miR-21, miR-155, and miR-27a are consistently upregulated in degenerated IVDs and are strongly associated with inflammatory activation and matrix catabolism [70,71,72]. MiR-21 has been shown to promote NP cell apoptosis and senescence by targeting phosphatase and tensin homologue (PTEN), thereby activating the PI3K/Akt and NF-κB pathways, which in turn enhance expression of MMPs and ADAMTS proteases [70]. Similarly, miR-155 amplifies inflammatory signalling by reinforcing NF-κB activity and suppressing negative regulators of cytokine signalling, leading to sustained production of IL-1β and TNF-α and accelerated ECM breakdown [72]. MiR-27a contributes to degeneration by directly repressing anabolic genes involved in collagen II and aggrecan synthesis while simultaneously upregulating catabolic enzymes via MAPK pathway activation [73,74]. Collectively, these pro-degenerative miRNAs establish a feed-forward loop in which inflammation, matrix degradation, and cellular dysfunction mutually reinforce one another. In contrast, several miRNAs exert protective effects in the IVD by suppressing inflammation and preserving ECM integrity. MiR-140, originally characterised in cartilage biology, is markedly downregulated in degenerated IVDs and plays a crucial role in maintaining proteoglycan homeostasis by inhibiting *ADAMTS-5* and *MMP-13* expression [73]. Restoration of miR-140 levels has been shown to reduce matrix degradation and improve NP cell viability in experimental models. MiR-146a functions as a negative feedback regulator of inflammation by targeting key adaptor molecules in the Toll-like receptor and NF-κB pathways, thereby limiting cytokine-induced catabolic responses [74]. Members of the miR-29 family suppress fibrotic and degenerative remodelling by regulating collagen turnover and inhibiting excessive ECM degradation [75,76]. These findings suggest that selective modulation of miRNA expression may be a promising strategy to rebalance anabolic and catabolic processes within the degenerated IVD.

Long non-coding RNAs constitute an additional, highly complex regulatory layer in IVD degeneration. LncRNAs are transcripts longer than 200 nucleotides that regulate gene expression through diverse mechanisms, including chromatin remodelling, transcriptional control, and post-transcriptional interactions with miRNAs and RNA-binding proteins. Several lncRNAs have been identified as key modulators of cell fate, inflammation, apoptosis, and senescence. LncRNA HOTAIR is significantly upregulated in degenerated NP tissue and promotes IVD degeneration by enhancing inflammatory signalling and ECM breakdown. Mechanistically, HOTAIR acts as a competing endogenous RNA (ceRNA), sequestering protective miRNAs such as miR-34a and miR-17, thereby releasing repression of pro-apoptotic and catabolic target genes [77,78]. This results in increased NP cell apoptosis, reduced matrix synthesis, and accelerated IVD degeneration. Similarly, *MALAT1* has been implicated in regulating inflammatory and oxidative stress responses in IVD cells. While some studies suggest context-dependent protective roles, aberrant *MALAT1* expression has been linked to dysregulated cell proliferation and altered ECM metabolism in degenerative conditions [79].

Other lncRNAs, including GAS5 and NEAT1, are increasingly recognised as regulators of cellular senescence and stress responses in IVD cells. GAS5 has been shown to promote apoptosis and inhibit proliferation by modulating glucocorticoid receptor signalling and p53-dependent pathways, contributing to reduced regenerative capacity of the IVD [80]. *NEAT1* participates in inflammasome activation and cytokine production, linking non-coding RNA regulation to chronic inflammation and the SASP signalling. Importantly, miRNAs and lncRNAs do not function in isolation but form highly interconnected regulatory networks. LncRNAs frequently act as molecular sponges for miRNAs, while miRNAs can regulate lncRNA stability and function, creating multilayered feedback loops that govern IVD cell behaviour. Disruption of these networks shifts the balance towards catabolism, inflammation, and senescence, thereby accelerating degeneration [81].

Taken together, these findings highlight post-transcriptional regulation by miRNAs and lncRNAs as a central molecular axis in IVD degeneration. Beyond their mechanistic relevance, non-coding RNAs hold significant promise as minimally invasive biomarkers for early disease detection and as therapeutic targets for disease-modifying interventions. Advances in RNA-based therapeutics, including miRNA mimics, antagomiRs (synthetic, chemically modified oligonucleotides designed to specifically inhibit miRNAs), and lncRNA-targeted approaches, may enable precise modulation of degenerative pathways and open new avenues for biologically driven treatment of DDD [68,81].

Increasing understanding of microRNAs and long non-coding RNAs has important translational implications for IVD degeneration [81]. Unlike conventional anti-inflammatory or analgesic therapies that primarily target downstream symptoms, non-coding RNAs act at upstream regulatory nodes controlling inflammation, extracellular matrix turnover, apoptosis, and cellular senescence, making them attractive candidates for disease-modifying interventions. Therapeutic strategies include miRNA mimics to restore protective miRNAs or inhibit pro-degenerative miRNAs, including miR-21 and miR-155 [68,81]. Preclinical studies demonstrate that targeted miRNA modulation reduces catabolic enzyme activity, suppresses inflammatory signalling, and enhances matrix synthesis in nucleus pulposus cells. Long non-coding RNAs offer additional opportunities due to their tissue-specific expression; targeting lncRNAs such as HOTAIR or *NEAT1* may disrupt pathogenic regulatory networks driving inflammation and senescence. Local delivery approaches, including injectable biomaterials and nanoparticle carriers, are particularly suitable for the avascular IVD environment. Collectively, ncRNA-based strategies represent a promising step towards precision therapies targeting the molecular drivers of IVD degeneration [68,78,79,80,81].

IVD degeneration is therefore not merely a passive consequence of mechanical wear, but a dynamic, biologically active process in which mechanical, biochemical, and cellular pathways interact. Recent discoveries in non-coding RNA regulation and senescence-targeted mechanisms significantly expand the molecular framework of IVD degeneration, providing new opportunities for biomarker development and disease-modifying therapy. Dysregulated cell–matrix signalling, chronic inflammation, oxidative stress, and maladaptive mechanical loading reinforce each other in a self-perpetuating cycle of degeneration. A deeper understanding of these interconnected mechanisms underpins the development of mechanobiologically informed therapeutic strategies aimed at restoring IVD integrity, modulating mechanical stress, and re-establishing tissue homeostasis [63,64].

## 5. Transition from Degeneration to Symptomatic Disease

**Figure 2:**
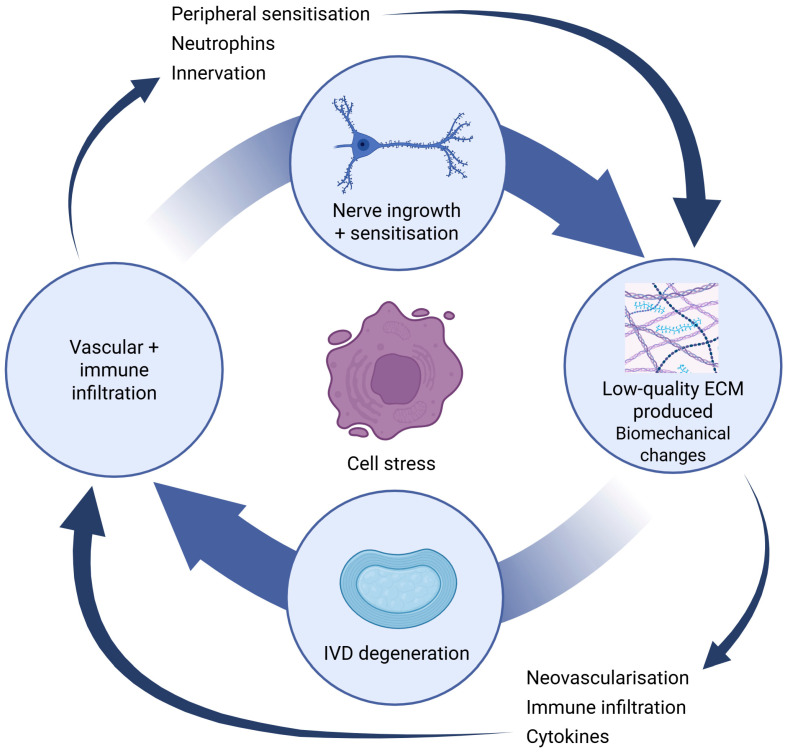
A schematic representation of proposed relationship between IVD degeneration, angiogenesis, immune cell infiltration, and nerve ingrowth and sensitization (adapted from reference [82]).

Importantly, not all IVD degeneration results in symptoms; many degenerated IVDs remain clinically silent. The transition to symptomatic disease (back pain, radiculopathy, spinal stenosis) involves several additional factors [1,2,26,52]. Fissuring of the AF allows proteoglycan fragments and proinflammatory cytokines to leak out, potentially stimulating nociceptors in the outer AF or adjacent ligaments. Ingrowth of sensory nerve fibres and neovascularisation in the IVD, which is normally immune-privileged and minimally innervated, are implicated in discogenic pain [26]. Mechanical instability and segmental hypermobility may also provoke pain through facet joint overload and ligamentous strain. Furthermore, imaging abnormalities alone often do not correlate directly with pain, suggesting that biochemical changes and microenvironmental factors (pH alteration, oxidative stress) play a key role in pain generation. Therefore, the concept of ‘IVD degeneration’ needs to be coupled with ‘pain generation’ to explain clinically symptomatic DDD [21,26,51]. A schematic representation of IVD degeneration, angiogenesis, immune cell infiltration and nerve ingrowth is depicted in Figure 2.

## 6. Structural and Functional Consequences

Degenerative changes within the IVD have profound structural and biomechanical repercussions that extend beyond the IVD itself, affecting adjacent ligaments, facet joints, and paraspinal musculature (Table 3). As IVD height decreases due to progressive dehydration and loss of proteoglycans, spinal segment stability is compromised. This results in abnormal motion patterns, altered load distribution, and progressive segmental instability, which together contribute to the cascade of secondary osteoarthritic changes in the motion segment [5,25].

Loss of IVD height increases axial loading on the facet joints, accelerating articular cartilage wear and subchondral bone remodelling, ultimately leading to facet joint osteoarthritis. Simultaneously, compensatory hypertrophy of the ligamentum flavum and capsular thickening further narrow the spinal canal, predisposing to central stenosis and neural compression [51]. Over time, these degenerative alterations can culminate in mechanical instability, spinal deformity, and progressive loss of mobility [4,5,6].

Pain in DDD arises from the interplay between mechanical deformation and biochemical sensitisation. Structural collapse and abnormal load transfer can directly irritate nerve roots, while inflammatory mediators released from degenerated IVD tissue, such as prostaglandins, interleukins (ILs), and TNF-α, promote nociceptor sensitisation. Moreover, neovascularisation and ingrowth of nociceptive nerve fibres into the normally aneural inner AF and NP, mediated by neurotrophic factors such as nerve growth factor (NGF), mark a key transition from structural degeneration to chronic pain [6,7,26,52]. Collectively, these structural, mechanical, and biochemical alterations transform the IVD from a load-bearing, shock-absorbing structure into a source of chronic inflammation and pain, underscoring the complex pathophysiology underlying DDD.

## 7. Degenerative Intervertebral Disc Disease in Clinical Practice

### 7.1. Emerging Diagnostic and Therapeutic Opportunities

Given expanding molecular insights, diagnostics and therapy for DDD are evolving from a purely structural paradigm towards stratified, mechanism-informed care. Advanced imaging techniques (diffusion-weighted MRI, quantitative T2 mapping) may improve phenotyping by capturing IVD hydration and microstructural integrity more precisely than conventional MRI. However, although these modalities are promising for detecting early biochemical changes, their routine clinical value remains limited by a lack of standardisation, variable thresholds across scanners, and uncertainty regarding how imaging biomarkers should guide treatment decisions in everyday practice [55].

On the therapeutic front, regenerative strategies such as intradiscal mesenchymal stem/stromal cell (MSC) delivery, gene-based modulation of catabolic pathways, scaffold-assisted repair, and senescence-targeting approaches are in preclinical or early clinical use [9,14]. Notably, the evidence base is currently strongest for biological plausibility and short-term symptomatic improvement, whereas durable structural regeneration and long-term disease modification are less consistently demonstrated. In other words, the field is moving from the question “can we influence IVD biology?” to the more clinically relevant question: “can we do so reliably, safely, and with sustained benefit that exceeds placebo and standard care?” These developments shift the paradigm from purely symptomatic treatment (analgesics, physiotherapy, surgical decompression) to biologically oriented approaches aimed at halting or reversing degeneration [4,5,6].

### 7.2. Regenerative and Molecular Therapies

Conventional conservative and surgical treatments for DDD primarily aim to alleviate pain and restore mechanical function to some extent, but do not reverse or halt the underlying degenerative cascade. This shortcoming has increased interest in regenerative and molecular therapies aimed at restoring extracellular matrix (ECM) homeostasis and reducing inflammation, thereby targeting the underlying pathophysiology of IVD degeneration. Consequently, regenerative medicine has emerged as a central focus of contemporary research, seeking to restore IVD structure, function, and homeostasis at the cellular and molecular levels. Experimental strategies include cell-based interventions using MSCs or NP-derived cells, growth factor delivery (*TGF-β, BMP-7*), gene or RNA-based modulation and PRP [67,89].

Among the most extensively investigated regenerative strategies is MSC therapy. MSCs derived from bone marrow, adipose tissue, or umbilical cord possess multipotent differentiation potential and a robust paracrine secretory profile [64,65]. When introduced into degenerated IVDs, MSCs can differentiate towards NP-like phenotypes and secrete trophic factors that stimulate resident cell proliferation, inhibit apoptosis, and promote ECM synthesis. In addition, MSCs exert strong immunomodulatory effects by downregulating proinflammatory cytokines such as TNF-α and IL-1β, thereby attenuating the chronic inflammatory milieu that drives degeneration. Early-phase clinical studies have demonstrated improvements in IVD hydration, as shown by T2-weighted magnetic resonance imaging, and patient-reported pain and function scores. A broad consensus exists that MSCs exert beneficial effects primarily through paracrine and immunomodulatory mechanisms, such as dampening pro-inflammatory cytokine signalling and altering the catabolic/anabolic balance, rather than through robust engraftment and replacement of native NP cells [40,67].

MSCs therapy demonstrated improvements in IVD hydration, which was proven in some experimental studies. In the prospective studies by Pettine et al., which evaluated autologous bone marrow concentrate containing MSCs, a ≥1-grade improvement in modified Pfirrmann score was observed in approximately 40% of treated IVDs at 12 months, with a reported mean improvement of about 0.3 grades per IVD, and greater improvements in subgroups receiving higher colony-forming unit doses [90]. These findings suggest partial structural stabilisation or modest reversal of degenerative changes rather than complete restoration of IVD morphology. Several early non-randomised studies also qualitatively reported increased T2-weighted signal intensity or water content at 12-month follow-up. However, absolute T2 signal intensity values or T2 mapping relaxation times were not consistently reported, limiting direct numerical comparison across trials [91]. A randomised controlled trial by Vadala et al., comparing intradiscal autologous bone marrow-derived MSC injections with a sham procedure, included up to three lumbar levels and reported intermediate clinical and radiological outcomes at six months [92]. The results included MRI assessment, although detailed T2 quantification has not yet been fully published. Orozco et al. remains one of the earliest human studies reporting improved IVD water content qualitatively on T2 MRI, but MRI quantification in other clinical trials has been limited or inconsistent. In contrast, the multicentre, randomised, and sham-controlled RESPINE trial using allogeneic bone marrow-derived MSCs applied a quantitative MRI-derived IVD fluid content index based on T2-weighted imaging. At 12 months, mean IVD fluid content changed by −5.1% in the MSC group versus −1.2% in the sham group, a difference that was not statistically significant [93]. At 24 months, a trend toward increased fluid content was observed in the MSC-treated group, but this also did not reach statistical significance. Similarly, no significant between-group differences in modified Pfirrmann grade progression were detected. Taken together, available clinical evidence indicates that MSC therapy is associated with modest and variable MRI-detected structural changes, including occasional Pfirrmann grade improvement or stabilisation and small relative changes in T2-weighted hydration metrics. These effects are less consistent than symptomatic improvements and have not yet demonstrated robust or durable IVD regeneration in controlled trials. MRI-based hydration improvements are therefore limited, heterogeneous, and primarily semi-quantitative [40,67,91,93]. However, across studies, structural outcomes are less consistent than symptomatic outcomes, and long-term durability and sustained disease modification have not been consistently demonstrated, likely due to heterogeneity in patient populations (discogenic pain versus mixed pain generators, Modic changes, and psychosocial confounding), differences in disease stage (with advanced degeneration limiting nutrient diffusion and cell survival), variability in MSC products (autologous versus allogeneic sources, dose, and manufacturing or expansion protocols), and technical constraints of intradiscal delivery (needle-related injury, leakage, uneven distribution, and limited retention). Overall, the weight of evidence supports MSC therapy as a potential symptom-modulating and biologically active intervention in carefully selected patients, but not yet as a reliably IVD-regenerating treatment. Accordingly, future trials should incorporate rigorous phenotyping, clinically meaningful controls, and predefined imaging and molecular biomarker endpoints to distinguish transient analgesic effects from true structural repair [40,67,89,94]. Long-term safety, optimal cell dosage, and delivery methods remain to be clarified in large randomised trials [40,67,89].

Advances in bioengineering and regenerative medicine have significantly reshaped therapeutic strategies for IVD degeneration, moving the field from symptomatic management towards biologically driven, structure-preserving, and disease-modifying approaches. Given the unique biomechanical environment and avascular nature of the IVD, effective therapies must address mechanical load-bearing, biological regeneration, and sustained local delivery of therapeutic agents. Recent progress in biomaterial design, controlled delivery systems, and early-phase clinical translation has brought several promising strategies closer to clinical application [40,67,94].

To optimise the microenvironment for cellular survival and matrix deposition, biomaterial-based scaffolds have been developed to replicate the mechanical and biochemical properties of the native IVD [40,67,95]. Biomaterials have been shown to improve cell retention, provide mechanical support, and create a permissive microenvironment for matrix synthesis. Preclinical studies generally support the concept that injectable hydrogels and composite scaffolds can improve short-term biomechanics and cellular viability. Injectable hydrogels, collagen composites, alginate microspheres, and nanofibre matrices provide three-dimensional support for cell retention, nutrient diffusion, and controlled release of bioactive molecules. Some scaffolds are being engineered as hybrid systems, incorporating growth factors or gene vectors to enhance regenerative signalling and integrate with host tissue. Current bioengineering efforts have focused on developing anisotropic and biphasic scaffolds that structurally and mechanically mimic the NP-AF interface. Biphasic scaffolds typically combine a hydrated, proteoglycan-mimetic core designed to replicate the viscoelastic properties of the NP with a circumferential, fibre-reinforced outer region that reproduces the tensile strength and anisotropy of the AF [95]. Electrospun nanofibrous scaffolds with angle-ply fibre orientation have been shown to closely mimic native AF lamellar architecture, supporting aligned cell growth and collagen deposition under physiological loading [96]. Injectable hydrogels, including alginate, gelatin methacrylate (GelMA), polyethylene glycol (PEG)-based hydrogels, and decellularised extracellular matrix (dECM)-derived materials, have gained attention due to their minimally invasive delivery and ability to conform to irregular IVD spaces [97,98]. Composite biomaterials integrating hydrogels with reinforcing fibres or microparticles further enhance mechanical stability while preserving nutrient diffusion and cell viability. These biomaterials can be engineered to respond dynamically to mechanical loading, supporting mechanotransduction pathways essential for IVD cell function and matrix homeostasis [93]. However, translation remains challenging because the degenerated IVD is a uniquely hostile niche: hypoxic, acidic, nutrient-poor, and mechanically loaded. The most consistent clinical rationale for biomaterials at present is adjunctive support, enhancing retention and survival of delivered cells or factors, rather than serving as a stand-alone regenerative solution. Key unresolved issues include implant integration, long-term mechanical behaviour under cyclical loading, and the risk of extrusion or altered endplate mechanics [58,67].

One of the major challenges in IVD regeneration is achieving sustained therapeutic activity within the IVD’s avascular and metabolically constrained environment. To address this, advanced delivery systems have been developed to enable controlled and prolonged release of growth factors and anti-inflammatory agents directly from biomaterial scaffolds. Encapsulation strategies using biodegradable microspheres and nanoparticles, commonly fabricated from poly(lactic-co-glycolic acid) (PLGA), chitosan, or lipid-based carriers, allow for controlled release kinetics ranging from days to several weeks [99]. Growth factors such as bone morphogenetic protein-2 (BMP-2), transforming growth factor-β (TGF-β), and insulin-like growth factor-1 (IGF-1) have been incorporated into these systems to stimulate anabolic matrix synthesis, promote IVD cell survival, and enhance regenerative responses [100,101]. Controlled release of BMP-2 from hydrogel–microsphere composites has demonstrated improved proteoglycan production while reducing the risk of ectopic ossification associated with bolus delivery [102,103]. Similarly, sustained delivery of anti-inflammatory agents, including corticosteroids, non-steroidal anti-inflammatory drugs, and emerging biological inhibitors, has been explored to attenuate chronic inflammation and catabolic signalling within the degenerated IVD. Hydrogel-based carriers enable localised drug retention and reduce systemic exposure, addressing safety concerns associated with repeated intradiscal injections [104]. Smart delivery platforms incorporating pH- or enzyme-responsive release mechanisms further allow therapeutic activation in response to the inflammatory microenvironment characteristic of IVD degeneration [105].

In parallel, gene and molecular therapies are increasingly recognised as precision-based strategies to modulate the catabolic–anabolic imbalance of the diseased IVD. There is broad consensus that targeting key molecular drivers of degeneration, such as matrix-degrading enzymes (MMPs, ADAMTS) and pro-inflammatory cytokines, represents a rational and mechanistically sound approach [32,57]. Preclinical studies consistently demonstrate that gene-editing technologies, including viral vector-mediated gene transfer and CRISPR/Cas9-based modulation, can suppress catabolic activity while enhancing the expression of anabolic growth factors such as TGF-β, BMP-7, and IGF-1, leading to improved extracellular matrix synthesis and partial restoration of IVD homeostasis. Preclinical models provide strong proof of concept, but clinical translation remains limited by delivery challenges, duration of expression, safety concerns, and regulatory barriers. Importantly, the IVD’s immune privilege may reduce systemic immune effects, yet it also complicates controlled dosing and long-term monitoring. At present, the consensus is that gene-based strategies are among the most biologically compelling but least clinically mature [32,33,57]. Similarly, RNA-based approaches using small interfering RNA and microRNAs show reproducible efficacy in vitro and in animal models by fine-tuning signalling pathways involved in matrix turnover, oxidative stress responses, and cellular senescence. However, despite this strong preclinical consensus, clinical translation remains limited and results are conflicting [30,39,62,63,64,65]. Key uncertainties persist regarding the safety of long-term gene modulation, off-target effects, durability of gene expression, and the efficiency of delivery within the hostile, avascular IVD microenvironment. Moreover, while anabolic and anti-catabolic molecular changes are frequently observed at the tissue level, it remains unclear whether these translate into sustained structural regeneration or meaningful long-term clinical benefit in humans [40,58,66,67]. Thus, although gene and molecular therapies are widely viewed as among the most biologically compelling regenerative strategies, the current evidence base supports their efficacy primarily at the experimental level, underscoring the need for carefully designed translational studies that integrate molecular endpoints with imaging and clinical outcomes [30,40,58,64,65,66,67,89,106,107].

Exosome-based therapies have also emerged as a promising acellular regenerative strategy, with growing consensus that extracellular vesicles derived from mesenchymal stem/stromal cells or NP cells can replicate many of the beneficial paracrine effects of cell-based therapies while avoiding the risks associated with live cell transplantation [89,108]. Preclinical studies consistently demonstrate that exosomes can deliver regenerative microRNAs, cytokines, and bioactive proteins that modulate inflammation, suppress catabolic signalling, and promote extracellular matrix synthesis, supporting the concept of biologically targeted IVD repair. Their acellular nature is widely regarded as advantageous in terms of safety, scalability, and regulatory feasibility. However, despite strong mechanistic plausibility and reproducible effects in vitro and in animal models, evidence regarding their therapeutic efficacy in humans remains limited and, in some cases, conflicting. Exosome-based therapies remain early in the translational pipeline, with unresolved issues regarding variability in exosome source and isolation methods, lack of standardised dosing and potency metrics, uncertainty regarding biodistribution and retention within the avascular IVD environment, and insufficient data on long-term structural and clinical outcomes. Exosome therapy should therefore be framed as an emerging platform with strong rationale but insufficient clinical proof [108,109]. Consequently, while there is broad agreement that exosome-based therapies represent a conceptual paradigm shift from symptomatic management towards biologically driven regeneration, their current evidentiary support remains predominantly preclinical. Robust translational and clinical studies integrating molecular, imaging, and patient-centred endpoints are required to determine whether these approaches can deliver durable, clinically meaningful benefits in DDD [89,106].

Encouraged by preclinical data, several cell-based and gene-based therapies for IVD degeneration have progressed into Phase I and II clinical trials [110]. Early-phase trials have demonstrated that intradiscal injection of autologous or allogeneic MSCs is generally safe and associated with improvements in pain scores, functional outcomes, and, in some cases, MRI-based indicators of IVD hydration [91,111]. Allogeneic progenitor cell products, such as nucleus pulposus-like cells derived from juvenile donors, have also shown promising early clinical results, with reported reductions in pain and stabilisation of IVD structure at short- to mid-term follow-up [112]. However, variability in cell survival, retention, and long-term efficacy remains a major limitation. Gene therapy approaches aim to induce sustained local production of anabolic or anti-inflammatory factors within the IVD. Viral and non-viral vectors delivering genes encoding TGF-β, BMPs, or inhibitors of catabolic enzymes have demonstrated regenerative potential in preclinical models. While clinical translation remains in early stages, ongoing Phase I trials are evaluating the safety of gene-modified cell therapies and direct gene delivery strategies. Collectively, these early clinical studies highlight the feasibility of biologically driven interventions for IVD degeneration while underscoring persistent challenges, including delivery efficiency, long-term durability, and patient selection. Integration of advanced biomaterials with cell and gene therapies is increasingly recognised as a critical step towards improving clinical outcomes and achieving functional IVD regeneration [112,113].

Another important option for DDD treatment is platelet-rich plasma (PRP). This approach is attractive because PRP is autologous and the procedure is minimally invasive. PRP contains growth factors that can stimulate anabolic processes in vitro. Some studies report meaningful pain reduction and functional improvement. However, the clinical literature remains inconsistent, plausibly due to poor standardisation (platelet concentration, leukocyte content, activation methods), variability in injection protocols, and heterogeneous inclusion criteria. Overall, the weight of evidence suggests PRP may benefit a subset of patients with discogenic pain, but it has not yet shown reliable structural regeneration, and the variability in preparation makes cross-study comparison difficult. Until protocols and patient selection are harmonised, PRP should be presented as promising but not definitive [114,115].

Taken together, current evidence supports regenerative and molecular therapies as promising, but not yet definitive, disease-modifying treatments. Although many studies report improvements in pain and function and demonstrate biologically plausible mechanisms (such as reduced inflammatory signalling and increased anabolic marker expression), clinical outcomes remain heterogeneous [30,58,65,66,67]. This heterogeneity likely reflects variability in patient selection, disease stage, product standardisation, injection techniques, and outcome measures. At present, the strongest evidence is for short-term symptomatic improvement in selected patients, while consistent and durable structural regeneration remains less conclusively established. Consequently, the field is increasingly moving towards combination approaches (such as combining cells and biomaterials, or optimising IVD biologics and mechanics) and towards stratified trials that align treatment choice with IVD phenotype and stage of degeneration. The main important questions for preclinical and clinical research are as follows: (I) DDD is a biologically active process involving inflammation, oxidative stress, and dysregulated ECM turnover; (II) regenerative strategies are most plausible when applied before end-stage structural collapse; and (III) patient selection and IVD phenotyping likely determine response more than the choice of biologic alone. Key areas of ongoing debate include whether reported symptomatic improvements reflect true structural IVD repair or instead represent transient anti-inflammatory or analgesic effects; the extent to which imaging changes, such as improved hydration or altered T2-weighted MRI signals, reliably predict long-term clinical outcomes; and which regenerative modality or combination of modalities can provide the most durable and clinically meaningful benefit [30,63,64,65,66,89,106,107,113]. Progress will likely depend on better trial design (including controls, blinding, and standardised products), longer follow-up, and integration of biomechanics, imaging biomarkers, and molecular endpoints [89,106].

## 8. Epidemiology, Diagnosis and Management of IVD

### 8.1. Epidemiological Context

Low back pain represents a major global health burden and one of the leading causes of disability worldwide. Its incidence varies widely across populations, ranking as the fifth most common reason for physician visits and affecting an estimated 7.6% to 37% of individuals at some point in their lives [1,2,31]. While most episodes of acute pain resolve spontaneously, approximately 10% of patients develop chronic or recurrent symptoms that significantly impair quality of life and productivity [1].

Degenerative changes in the IVD often begin insidiously, with structural and biochemical alterations detectable even in adolescence. Epidemiological studies have demonstrated early signs of IVD degeneration in up to 20% of adolescents and in nearly 90% of elderly individuals, highlighting the progressive and cumulative nature of the process [4,5,6]. Notably, many individuals remain asymptomatic despite advanced degeneration, underscoring the complex relationship between radiological findings and clinical manifestations [31,42].

### 8.2. Clinical Manifestations

The clinical presentation of DDD is highly variable and depends on the degree, location, and pattern of degeneration, as well as secondary changes affecting surrounding spinal structures. In its early stages, IVD degeneration may remain asymptomatic and is often detected incidentally through imaging performed for unrelated reasons [13,116]. As structural integrity declines and mechanical load distribution becomes altered, patients typically develop axial spinal pain, most often chronic low back pain, characterised by stiffness, reduced flexibility, and discomfort that worsens with prolonged sitting, bending, or lifting. This pain is primarily mechanical, reflecting abnormal motion and microinstability within degenerated motion segments [42,43].

With progressive IVD collapse and annular fissuring, nerve root irritation or compression may occur, leading to radicular symptoms. Patients may experience radiating pain, paraesthesia, or numbness following a dermatomal pattern, commonly described as sciatica in the lumbar spine or cervicobrachial neuralgia in the cervical region [13]. In severe cases, motor weakness and reflex changes may develop, indicating neural compromise. Discogenic pain may also be referred to distant regions through shared segmental innervation, complicating clinical localisation. Additionally, chronic nociceptive stimulation can lead to central sensitisation, amplifying pain perception even in the absence of clear structural impingement [2,3,4,5,6,7,107].

Proinflammatory mediators released from degenerated IVD tissue, such as IL-1β, TNF-α, and prostaglandins, contribute to biochemical sensitisation of nociceptors and may explain why pain intensity does not always correlate with radiographic findings. The ingrowth of nociceptive nerve fibres and neovascularisation into the normally aneural inner AF and NP further perpetuate chronic pain. Paraspinal muscle spasm, reduced core stability, and compensatory postural adaptations may develop secondarily, further limiting function [7,8,9,13,14,16]. Beyond localised pain, patients with advanced DDD often report functional impairment, including diminished range of motion, morning stiffness, and activity intolerance, which significantly affect quality of life. In lumbar disease, prolonged degeneration may lead to spinal canal stenosis, presenting with neurogenic claudication, pain, numbness, or weakness in the lower limbs that worsens with walking and improves with flexion. Cervical involvement may manifest with myelopathic symptoms such as gait disturbance or hand clumsiness due to spinal cord compression. Overall, the clinical manifestations of DDD reflect a complex interplay of structural degeneration, mechanical dysfunction, inflammatory sensitisation, and neural adaptation. Recognition of this multifactorial nature is essential for accurate diagnosis, patient stratification, and individualised therapeutic management [18,31,42,43,83,107,113,116,117,118,119].

### 8.3. Diagnostic Evaluation

The clinical diagnosis of DDD relies on the integration of clinical assessment, imaging studies, and, in select cases, functional and biochemical evaluations [7,9,13]. A comprehensive patient history and physical examination remain the cornerstone of diagnosis. Clinicians should assess pain characteristics, location, duration, and aggravating or relieving factors, alongside neurological symptoms such as sensory loss, motor weakness, or reflex changes. Physical examination often reveals paraspinal tenderness, limited spinal mobility, and pain exacerbated by axial loading or flexion–extension manoeuvres, suggestive of segmental instability. However, clinical findings alone are not pathognomonic and must be correlated with imaging to confirm degenerative changes [42,43].

Magnetic resonance imaging (MRI) is considered the gold standard for evaluating IVD degeneration due to its superior soft-tissue contrast and ability to visualise IVD hydration, morphology, and surrounding neural structures. Typical MRI features of DDD include loss of T2 signal intensity indicating dehydration, reduction in IVD height, annular fissures, bulging or herniation, and Modic changes in adjacent vertebral endplates reflecting marrow inflammation or sclerosis. Quantitative MRI techniques, such as T2-weighted mapping and diffusion tensor imaging, have emerged as valuable tools for assessing the biochemical composition and microstructural integrity of the IVD, offering potential for early detection of degeneration before structural deformities appear [18,117,118].

Computed tomography (CT) provides detailed visualisation of osseous changes, including CEP sclerosis, osteophyte formation, and facet joint degeneration, and is often used in surgical planning or when MRI is contraindicated [18]. Plain radiographs can demonstrate IVD space narrowing, vacuum phenomena, and spinal alignment abnormalities but are limited in soft-tissue evaluation. Discography, though controversial, may be used selectively to identify discogenic pain when imaging findings are inconclusive, although it carries risks such as infection or acceleration of degeneration [116].

Emerging biochemical and molecular biomarkers, including markers of matrix degradation (type II collagen fragments, aggrecan breakdown products) and inflammatory mediators in serum or IVD aspirates, are being investigated as adjuncts for diagnosis and monitoring of disease progression. Additionally, advances in artificial intelligence and radiomics hold promise for improving diagnostic accuracy by integrating imaging patterns with clinical and genetic data [83,119,120].

Accurate diagnostic evaluation of DDD ultimately requires a multifaceted approach that combines structural imaging, clinical context, and, increasingly, molecular insights. Such an integrated framework not only facilitates early detection but also supports personalised therapeutic decision-making and prognostic assessment [83,116].

### 8.4. Therapeutic Approaches

The management of DDD is multifaceted, aiming to alleviate pain, restore function, and prevent further degeneration [4,7]. Treatment strategies are typically guided by symptom severity, the extent of structural degeneration, and the presence of neurological compromise. As DDD is a chronic and progressive condition, a stepwise approach—beginning with conservative measures and progressing to invasive interventions when necessary—is generally recommended (Table 4). Conservative management remains the cornerstone of initial therapy [20,120]. It includes physical rehabilitation, pharmacological treatment, and lifestyle modification. Structured physical therapy focuses on strengthening paraspinal and core muscles, improving flexibility, and enhancing postural control to stabilise degenerated segments and reduce mechanical strain. Lifestyle interventions, particularly weight reduction, smoking cessation, and ergonomic adjustments, address modifiable risk factors and help slow disease progression. When these measures are insufficient to relieve pain, more aggressive strategies, such as medications and surgery, may be considered. Current treatment guidelines for chronic back pain and DDD primarily focus on symptom relief through medications or surgery. While these approaches can reduce pain, they do not halt or reverse IVD degeneration. Consequently, there is growing interest in new therapeutic strategies that target underlying disease mechanisms, though further research is needed [24,86,88].

For medical management of DDD, non-steroidal anti-inflammatory drugs (NSAIDs), muscle relaxants, and short courses of analgesics are commonly used to manage pain and inflammation. NSAIDs inhibit cyclooxygenase (COX) enzymes responsible for prostaglandin synthesis and inflammation [121]. Although the limited vascular supply in the IVD may reduce their local effectiveness, NSAIDs generally provide short-term pain relief and are easily accessible. However, they may increase the risk of gastrointestinal ulcers, cardiovascular complications, and renal impairment [86,87,121]. Prescription opioids are used when pain cannot be controlled with over-the-counter medications. They mimic endogenous opioid peptides and activate G-protein–coupled receptor (GPCR) signalling to suppress neurotransmitter release and reduce pain transmission. While effective, opioids carry significant risks, including addiction and chronic dependence. Patients using opioids for chronic low back pain (LBP) have been shown to develop long-term use at higher rates than those treated for other musculoskeletal conditions. Epidural corticosteroid injections offer another option for short-term relief by reducing inflammation through inhibition of arachidonic acid release and COX activity [122]. Ongoing trials are evaluating whether specific injection routes improve outcomes. However, steroids can cause systemic side effects and do not provide sustained pain relief, limiting their long-term usefulness. Several therapies traditionally used for bone diseases or systemic disorders are being explored for discogenic pain, such as pamidronate (a bisphosphonate used to inhibit osteoclast activity, which shows potential for reducing pain due to its effects on bone turnover), and abaloparatide (although used in osteoporosis, it has been shown to reduce IVD degeneration in animal models). Alpha-2-macroglobulin (A2M) is also being investigated for its ability to inhibit proteases that degrade cartilage and produce fibronectin–aggrecan complex (FAC), a degradation product implicated in DDD-related pain. Clinical trials aim to determine whether concentrated autologous A2M can slow IVD degeneration [122,123].

**Figure 3:**
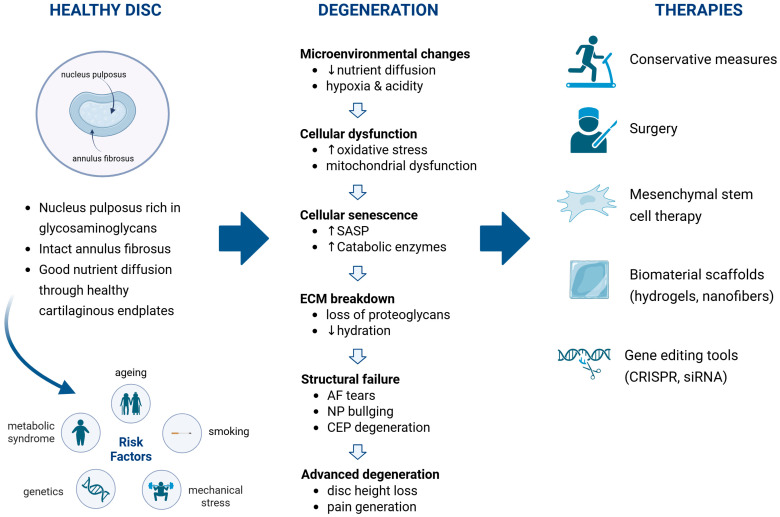
Overview of IVD homeostasis, degeneration, and therapeutic strategies. A healthy IVD is characterized by a glycosaminoglycan-rich NP, an intact AF, and efficient nutrient diffusion through CEP. Ageing and risk factors, including genetic predisposition, metabolic syndrome, smoking, and mechanical stress, contribute to progressive IVD degeneration. Microenvironmental alterations such as reduced nutrient diffusion, hypoxia, and acidosis induce cellular dysfunction, oxidative stress, mitochondrial impairment, and cellular senescence, leading to increased catabolic activity and ECM degradation. These changes result in structural failure and advanced degeneration associated with IVD height loss and pain. Current and emerging therapeutic approaches range from conservative treatment and surgery to regenerative strategies, including mesenchymal stem cell therapy, biomaterial scaffolds, and gene-based interventions. *Abbreviations: NP, nucleus pulposus; AF, annulus fibrosus; CEP, cartilaginous endplate; ECM, extracellular matrix; SASP, senescence-associated secretory phenotype; CRISPR, clustered regularly interspaced short palindromic repeats; siRNA, small interfering RNA*.

When conservative therapies fail to achieve adequate symptom control, surgery is generally reserved for patients with severe chronic LBP unresponsive to conservative therapy. Discectomy and decompression procedures aim to relieve neural compression in cases of herniation or stenosis, while spinal fusion is employed to stabilise unstable motion segments and alleviate mechanical pain [84,89,124]. Spinal fusion involves removing the degenerated IVD, inserting a cage to maintain IVD height, and stabilising the spine with screws or rods. The goal is to permanently fuse adjacent vertebrae and reduce painful motion. Minimally invasive techniques reduce tissue damage but require specialised equipment and longer operative times. Although fusion may relieve pain in some patients, it decreases spinal mobility and may accelerate degeneration of adjacent IVDs. Some patients continue to require opioid therapy post-surgery. Total disc replacement removes the entire IVD and replaces it with an artificial device composed of metal endplates and a plastic core [85]. This procedure aims to preserve motion, unlike fusion. Trials comparing disc replacement with fusion show improvements in short-term pain, disability, and quality of life, though differences are not always clinically significant. In randomised comparisons, both approaches improve pain and disability, with total disc replacement showing small between-group differences in some trials. For example, in an FDA IDE randomised trial comparing ProDisc-L to circumferential fusion, mean Oswestry Disability Index (ODI) scores improved from 63 to 32 at 24 months in the prosthesis group versus 62 to 37 in the fusion group, while pain improvement on the VAS scale was 4 versus 3, respectively [126]. In another randomised trial by Sasso et al., mean ODI decreased from 62 to 6 at 24 months after total disc replacement versus 58 to 12 after fusion, and mean VAS decreased from 8 to 1 versus 8 to 2, respectively, again indicating broadly similar improvements with numerically slightly better scores in the total disc replacement arm [126,127]. Overall, these RCTs support that total disc replacement and fusion can both yield meaningful improvements in ODI and VAS in selected patients, while the magnitude of the average between-group difference is generally small and variably significant across studies. Ongoing trials are evaluating next-generation artificial IVDs. Despite frequent use, evidence suggests that surgery offers limited long-term benefit for many patients [35,82,85,88,89,106]. In recent years, there has been growing interest in biological and regenerative therapies targeting the underlying pathophysiology of IVD degeneration [110,128]. A graphical summary connecting IVD anatomy, degenerative processes and therapy is summarised in Figure 3.

Experimental strategies include the application of platelet-rich plasma (PRP), gene therapy, growth factor delivery (TGF-β, BMP-7), and cell-based interventions using MSCs or NP-derived cells to restore extracellular matrix synthesis and IVD hydration [35,85]. PRP is being investigated as a minimally invasive treatment for discogenic LBP. Derived from the patient’s own blood, PRP is rich in growth factors such as PDGF, TGF-β, VEGF, and IGF-1, which have been shown in vitro and in animal models to promote extracellular matrix synthesis and IVD cell proliferation. Some studies report restoration of IVD height and improved matrix production. However, human data remain inconsistent, and challenges include the lack of standardised PRP preparation methods and uncertainty regarding optimal delivery techniques. Stem cell-based therapies show promise for regenerating IVD tissue. The aim is to implant viable cells into the degenerated IVD to restore structure and function. Current trials are investigating bone marrow-derived MSCs, umbilical cord MSCs, and allogeneic MSCs. Research is also exploring notochordal cells and induced pluripotent stem cells due to their closer resemblance to native IVD cell populations. Despite encouraging early results, a major challenge is poor cell survival within the harsh, nutrient-deprived microenvironment of the degenerated IVD [24,125]. Further work is needed to determine optimal cell types, delivery systems, and supportive biomaterials. Although early preclinical and clinical trials show promising results, challenges such as limited cell survival, immunogenicity, and delivery methods remain. Effective management of DDD ultimately requires an individualised, multimodal approach that integrates mechanical stabilisation, pain modulation, and biological restoration. Future therapeutic paradigms will likely combine conventional treatments with regenerative and molecular interventions, aiming not only to relieve symptoms but also to modify the course of IVD degeneration itself [24,82,88].

## 9. Translational Challenges from Animal Models to Human Disease

Animal models have been indispensable for elucidating the molecular, cellular, and biomechanical mechanisms underlying IVD degeneration; however, a substantial translational gap persists between preclinical success and outcomes observed in human clinical trials. Widely used experimental models, including needle puncture injury, enzymatic degradation, mechanical overload, and genetically modified rodents, have provided critical insights into ECM breakdown, inflammatory cytokine signalling, oxidative stress, mechanotransduction, and cellular senescence pathways implicated in DDD. These models have been particularly valuable for establishing causal relationships between catabolic signalling (IL-1β, TNF-α, NF-κB), matrix-degrading enzymes (MMPs, ADAMTS), and progressive loss of IVD hydration and biomechanical function [16,21,22,23,110].

Most animal models reproduce acute or artificially accelerated degeneration in young, otherwise healthy IVDs with intact vascular supply and metabolic reserve. This contrasts sharply with human DDD, which is a slow, age-associated, multifactorial process unfolding over decades and strongly influenced by reduced endplate permeability, impaired nutrient diffusion, chronic low-grade inflammation, metabolic disorders, and cumulative mechanical loading [16,17,20]. As a result, biological responses observed in animal models, particularly regenerative responses, may overestimate the reparative potential of the human IVD. These differences have major implications for the translation of regenerative therapies. In preclinical studies, interventions such as MSC transplantation, growth factor delivery (TGF-β, BMP-7), gene therapy targeting catabolic enzymes, and biomaterial scaffolds frequently demonstrate robust restoration of proteoglycan content, IVD height, and hydration, as well as suppression of inflammatory signalling [22,58,110]. In contrast, human clinical trials have generally reported modest and heterogeneous effects, with improvements more consistently observed in pain and function rather than durable structural regeneration. Several biological and biomechanical barriers likely contribute to this divergence. These include poor survival and retention of transplanted cells within the hypoxic, acidic, and nutrient-deprived human IVD microenvironment; sclerotic or calcified cartilaginous endplates that severely limit nutrient diffusion; and the reduced anabolic capacity of senescent cells in advanced degeneration [17,19,20,110].

A further translational challenge arises from the mismatch between experimental and clinical outcome measures. Animal studies typically assess efficacy using histological scoring, biochemical quantification of proteoglycans and collagen, gene expression profiles, and high-resolution imaging of IVD morphology. In contrast, human trials necessarily prioritise patient-reported outcomes (pain, disability, quality of life) and semi-quantitative MRI grading systems such as the Pfirrmann classification. While clinically meaningful, these measures may not capture subtle biological changes, early matrix stabilisation, or delayed structural benefits, complicating interpretation of negative or equivocal trial results. Conversely, improvements in IVD structure observed in animals do not always translate into pain reduction in humans, reflecting the complex and multifactorial nature of discogenic pain, which involves neural ingrowth, inflammatory sensitisation, central pain processing, and psychosocial factors [5,26,57,88]. Importantly, most animal models represent early or moderate degeneration with preserved IVD architecture, whereas many human trials enrol patients with advanced disease, characterised by annular fissuring, endplate pathology, IVD height collapse, and segmental instability. In such contexts, the biological reversibility of degeneration is likely limited. This highlights the need for stage-specific therapeutic strategies, in which regenerative or molecular interventions are applied earlier in the disease course, while advanced degeneration may require combined biological and mechanical approaches or remain best managed surgically [16,82,89].

Taken together, the limited clinical translation of regenerative therapies does not necessarily indicate biological inefficacy, but rather reflects fundamental differences between experimental models and human disease biology. Bridging this gap will require improved preclinical models that better recapitulate ageing, metabolic dysfunction, and endplate pathology; validation of translational biomarkers linking molecular changes to clinical outcomes; harmonisation of structural and functional endpoints across studies; and careful patient stratification based on disease stage and biological phenotype. Addressing these challenges is essential for transforming promising experimental therapies into reliable, disease-modifying treatments for human DDD [82,128].

Over the past two decades, substantial progress has been made in elucidating the molecular and cellular mechanisms underlying IVD degeneration. The current body of knowledge recognises DDD as an active, biologically driven process characterised by extracellular matrix breakdown, chronic low-grade inflammation, oxidative stress, cellular senescence, and altered mechanotransduction, rather than a passive consequence of mechanical wear alone. These concepts are well supported by in vitro studies and a wide range of animal models, and have reshaped contemporary thinking about potential disease-modifying interventions [82,89].

However, despite strong mechanistic coherence at the experimental level, translation of these insights into effective clinical therapies has been limited. A critical evaluation of this translational gap reveals that many promising therapeutic strategies succeed in preclinical models yet show only modest or inconsistent benefits in human trials. This discrepancy reflects fundamental differences between experimental systems and the biological, mechanical, and clinical realities of human IVD degeneration [82,110,128].

Animal models have been indispensable for defining pathogenic pathways and testing regenerative concepts. Commonly used models, including needle puncture injury, enzymatic matrix degradation, mechanical overload, and genetically modified rodents, reliably reproduce key molecular features of degeneration, such as increased inflammatory cytokine signalling, upregulation of matrix-degrading enzymes, loss of proteoglycans, and IVD dehydration. These models have convincingly demonstrated that targeted interventions (MSCs, growth factors, gene modulation, and biomaterials) can restore matrix composition, improve IVD height, and suppress catabolic signalling under controlled conditions [89,128]. Nevertheless, these models typically induce acute or accelerated degeneration in young, metabolically healthy IVDs, which contrasts sharply with human DDD. In patients, IVD degeneration is a slow, age-associated, and multifactorial process that develops over decades in an avascular tissue with limited nutrient supply, progressive endplate sclerosis, systemic metabolic influences, and cumulative mechanical loading. As a result, regenerative responses observed in animals may overestimate the reparative capacity of the human IVD, particularly in advanced disease [110,125,128]. This divergence is especially evident in the clinical translation of regenerative therapies. While preclinical studies frequently report robust restoration of IVD hydration and matrix integrity, human trials more consistently demonstrate improvements in pain and function rather than durable structural regeneration. Several biological barriers likely contribute to this outcome, including poor survival and retention of transplanted cells in the hostile human IVD microenvironment, impaired nutrient diffusion across calcified cartilaginous endplates, and the reduced anabolic potential of senescent IVD cells. These constraints are largely absent or attenuated in most animal models, limiting their predictive value for clinical efficacy [82,89,128].

A further limitation lies in the mismatch between experimental and clinical endpoints. Animal studies typically rely on histological, biochemical, and molecular outcomes that directly quantify matrix restoration and cellular behaviour. In contrast, human trials prioritise patient-reported outcomes and semi-quantitative imaging metrics, which may not capture subtle biological effects or early structural stabilisation. Conversely, improvements in IVD structure do not necessarily translate into pain relief, reflecting the complex, multifactorial nature of discogenic pain involving neural ingrowth, inflammatory sensitisation, central pain processing, and psychosocial factors. This disconnect complicates interpretation of both positive and negative clinical trial results [9,13,82,89]. Importantly, many regenerative strategies are tested preclinically in models representing early or moderate degeneration with preserved IVD architecture, whereas clinical trials often enrol patients with advanced disease, characterised by annular fissuring, IVD height collapse, endplate pathology, and segmental instability. In such cases, the biological reversibility of degeneration may be inherently limited. This highlights the need to reconceptualise regenerative therapies not as universal solutions, but as stage-dependent interventions that may be most effective when applied early in the degenerative cascade [13,16,110,125,128].

Taken together, these observations suggest that the limited clinical success of regenerative and molecular therapies is not primarily due to flawed biological concepts, but rather to a misalignment between experimental models and the complexity of human disease. Bridging this translational gap will require the development of preclinical models that better incorporate ageing, metabolic dysfunction, and endplate pathology; improved alignment of preclinical and clinical endpoints; validation of translational biomarkers; and careful patient stratification based on disease stage and biological phenotype [89,110,128].

In this context, future therapeutic progress in DDD is likely to depend less on identifying novel molecular targets and more on integrating existing biological insights with realistic clinical frameworks. This approach moves the field beyond descriptive pathogenesis and uncritical enthusiasm for emerging therapies, toward a more rigorous, translationally grounded strategy capable of delivering meaningful disease modification in human patients [84,86,110,125].

## 10. Future Directions

Despite substantial progress in elucidating the molecular, cellular, and biomechanical mechanisms underlying IVD degeneration, the translation of these insights into durable, disease-modifying therapies remains limited [40,67,115]. Much of the current literature is characterised by descriptive molecular studies, heterogeneous preclinical models, and early-phase clinical trials with variable methodologies and short follow-up periods. As a result, promising biological concepts have not yet converged into reproducible, standardised clinical strategies capable of altering the natural history of DDD [4,6,7,67,91].

To advance the field beyond proof-of-concept toward clinically meaningful intervention, future research must shift from isolated mechanistic observations to integrated, hypothesis-driven approaches that explicitly link biological targets with functional and structural outcomes. This will require improved alignment between preclinical models and human disease, more rigorous clinical trial design, and a stronger emphasis on longitudinal assessment of both biological and mechanical endpoints [13,19]. In particular, regenerative and molecular therapies must be evaluated not only for short-term symptom relief, but also for their ability to stabilise IVD structure, preserve biomechanical function, and delay or prevent downstream spinal degeneration [89,107]. Another critical limitation is the lack of consensus regarding what constitutes therapeutic success in IVD degeneration [30,64]. Expectations of complete IVD regeneration may be unrealistic in many clinical contexts; instead, future strategies should prioritise achievable goals such as slowing degeneration, restoring partial matrix homeostasis, preventing further height loss, or reducing the inflammatory and nociceptive milieu within the IVD. Establishing such realistic and measurable objectives is essential for rational trial design, regulatory approval, and clinical adoption [13,19,21,67].

Finally, the inherently multifactorial nature of IVD degeneration demands a departure from one-dimensional treatment paradigms. Effective future therapies will likely require multimodal integration of biological, mechanical, and lifestyle-based interventions, tailored to disease stage and patient phenotype. Achieving this will depend on the development of validated biomarkers, standardised intervention protocols, and long-term outcome datasets that collectively enable precision medicine approaches in DDD [4,84,85]. On this basis, several priority areas for future research and clinical translation can be identified:

(I) Standardisation of cell-based and regenerative therapies: A critical barrier to interpreting and comparing outcomes of MSC-based therapies is the lack of standardisation across trials. Future studies must define and harmonise key parameters, including cell source (autologous versus allogeneic), tissue origin (bone marrow, adipose tissue, umbilical cord), cell dose, expansion protocols, and viability criteria at the time of injection [21,64,89]. Reporting of MSC phenotype (surface markers, senescence status, secretory profile) should be mandatory, as accumulating evidence suggests that therapeutic effects are largely paracrine and immunomodulatory rather than driven by long-term engraftment. Equally important is the standardisation of delivery techniques, including needle gauge, injection volume, injection pressure, and the number of treated levels, as these factors influence IVD injury, leakage, and cell retention. The integration of biomaterial carriers or hydrogels should be systematically evaluated to improve cell survival in the hypoxic, nutrient-poor IVD microenvironment. Without such harmonisation, meaningful meta-analysis and regulatory progression will remain difficult [13,64,65,106].

(II) Validation of biomarkers for patient stratification and monitoring: A major unmet need in DDD is the absence of validated biomarkers that identify patients most likely to benefit from biological or regenerative interventions [19,37,38,59]. Future research should prioritise biomarker qualification, rather than exploratory reporting alone. Candidate biomarkers include MRI-based quantitative measures (T2 mapping, diffusion metrics), biochemical markers of matrix turnover (aggrecan and type II collagen degradation products), and inflammatory mediators such as IL-1β, TNF-α, and IL-6 in serum or IVD aspirates. Critically, biomarkers must be evaluated longitudinally and correlated with both structural outcomes and clinically meaningful endpoints (pain, function, work capacity). This will allow separation of transient analgesic effects from true biological modification. Multimodal approaches combining imaging, molecular profiling, and clinical phenotyping, potentially supported by artificial intelligence and radiomics, represent a promising direction for precision medicine in DDD [36,37,58,129].

(III) Defining appropriate clinical endpoints for regeneration: Many regenerative trials continue to use broad patient-reported outcomes as primary endpoints, despite the stated aim of structural repair. Future trials should adopt predefined hierarchical endpoints that incorporate both quantitative imaging metrics and clinical outcomes [91,115]. Importantly, expectations regarding the magnitude and timeline of structural change must be realistic; IVD regeneration is likely to be slow and partial rather than complete. Consensus is needed on what constitutes meaningful structural benefit, such as stabilisation of Pfirrmann grade, prevention of further IVD height loss, or modest improvements in hydration indices, rather than complete restoration of a normal, intact, healthy IVD. Establishing these benchmarks will improve trial design, power calculations, and interpretation of negative or equivocal results [90,92,115].

(IV) Addressing gaps in long-term surgical outcomes: Although surgical interventions such as spinal fusion and total disc replacement are widely performed, long-term comparative data beyond 10 to 15 years remain sparse, particularly regarding adjacent segment degeneration, reoperation rates, opioid dependence, and functional durability [4,84]. Future studies should prioritise prospective registries and extended follow-up of randomised cohorts, rather than relying solely on short-term efficacy. For total disc replacement specifically, there is a need for systematic evaluation of implant wear, device migration, facet joint degeneration, and revision complexity, as well as identification of patient subgroups most likely to benefit from motion-preserving strategies. These data are essential to inform evidence-based surgical decision-making and cost-effectiveness analyses [13,84,85,124].

(V) Integration of mechanical and biological therapeutic strategies: IVD degeneration arises from the interaction of mechanical overload and biological dysfunction; therefore, future therapies should not address these domains in isolation [26,51]. Regenerative approaches are unlikely to succeed without addressing segmental biomechanics, posture, muscle function, and load distribution. Combined strategies, such as biological interventions paired with targeted rehabilitation, mechanical unloading, or motion-preserving stabilisation, should be systematically explored. Similarly, preclinical models and clinical trials should better account for endplate health and nutrient transport, as endplate sclerosis and vascular compromise critically limit regenerative capacity. Therapies targeting endplate permeability and vertebral–IVD crosstalk represent an underexplored but potentially transformative area [1,3,13,94,108].

(VI) Regulatory, ethical, and translational considerations: Successful translation of regenerative therapies will require alignment with regulatory frameworks. Clear definitions of manufacturing standards, quality control, and safety endpoints are essential, particularly for advanced therapies such as gene editing, exosome-based treatments, and senolytics. Early engagement with regulatory authorities and incorporation of adaptive trial designs may accelerate safe clinical translation [39,62,63,110].

In summary, the field must shift from heterogeneous proof-of-concept studies toward standardised, biomarker-driven, and longitudinally designed research programmes. Addressing these actionable priorities will be essential to transform emerging molecular and regenerative insights into reliable, disease-modifying therapies for DDD [27,110,125].

## 11. Limitations of Our Review

This article is a narrative review, and its limitations should be interpreted in light of its intended scope and objectives. Unlike systematic reviews or meta-analyses, this work was not designed to comprehensively capture all available publications using a predefined search strategy or formal quality assessment framework. Instead, the review aims to provide an integrative, concept-driven synthesis of key biological, molecular, and clinical aspects of IVD degeneration. As such, some selection bias is unavoidable, and the cited literature may not fully represent all existing evidence, particularly studies with negative or contradictory findings.

Another limitation stems from the heterogeneity of the underlying literature. Many mechanistic insights discussed in this review are derived from in vitro systems or animal models, which, while essential for elucidating molecular pathways, cannot fully replicate the complex biomechanical loading, avascularity, and metabolic constraints of the human IVD. Consequently, caution is necessary when extrapolating experimental findings to human pathology or clinical outcomes.

Furthermore, although emerging regenerative and molecular therapies are highlighted as promising avenues, current clinical evidence supporting these approaches remains limited. Most data come from preclinical studies or early-phase clinical trials with small cohorts, heterogeneous methodologies, and relatively short follow-up periods. Therefore, definitive conclusions regarding long-term efficacy, safety, and clinical implementation cannot yet be drawn, and the discussion of these therapies should be regarded as exploratory rather than prescriptive.

Finally, this review does not attempt a quantitative comparison of treatment modalities or a formal assessment of clinical effectiveness or cost–benefit ratios. The complex and incompletely understood relationship between structural IVD degeneration, imaging findings, and pain generation remains a significant challenge in the field, and is reflected in the variability and sometimes conflicting conclusions of the studies discussed.

## 12. Conclusions

In conclusion, DDD and back pain represent a complex, multi-layered disease process rooted in mechanical, genetic, molecular, nutritional, and environmental dysfunction. Although the IVD was long regarded as a passive structural element, modern science has shown it to be a metabolically active tissue whose failure reflects both systemic and local derangements. While traditional therapies focus on pain reduction and restoring mobility, emerging molecular and regenerative techniques offer the potential to reverse or slow IVD degeneration at its biological roots. Despite considerable progress in understanding IVD structure, degeneration, and pain mechanisms, critical gaps remain. These include identifying reliable diagnostic biomarkers, understanding early degeneration before structural damage occurs, and translating molecular therapies into safe, effective clinical practice. Recent research has increasingly focused on regenerative and molecular approaches that target the pathophysiology of the disease rather than its consequences. The main therapeutic goal is to restore IVD structure and function by promoting ECM synthesis, inhibiting catabolic enzymes, and improving cellular viability within the IVD microenvironment. Future research should aim to integrate molecular biology, materials science, and clinical translation to establish standardised protocols for cell-based and gene-based therapies. Understanding the intricate molecular mechanisms of IVD ageing will be essential for developing truly restorative treatments capable of addressing the global burden of spinal degenerative disease.

## Figures and Tables

**Table 3:** Structural and functional consequences of IVD degeneration.

Category	Key Changes	Consequences/Effects	References
**Structural degeneration**	Dehydration and proteoglycan loss Decreased IVD height	Compromised spinal stability Abnormal motion patterns Altered load distribution Segmental instability	[8,13,14,16]
**Facet joint changes**	Increased axial load on facets Articular cartilage wear Subchondral bone remodelling	Facet joint osteoarthritis	[1,9,16,26]
**Ligamentous changes**	Ligamentum flavum hypertrophy Capsular thickening	Spinal canal narrowing Central stenosis Neural compression	[83,84,85]
**Mechanical outcomes**	Progressive degenerative alterations Instability and deformity	Reduced mobility Worsening of mechanical dysfunction	[1,16,51]
**Biochemical sensitisation**	Release of inflammatory mediators (prostaglandins, ILs, TNF-α)	Nociceptor sensitisation Increased inflammatory pain	[40,86,87]
**Neural changes**	Neovascularisation Nerve ingrowth into inner AF and NP (via NGF)	Transition from structural degeneration to chronic pain	[5,82,88]
**Overall pathophysiology**	Combination of structural, mechanical, and biochemical changes	IVD becomes a source of chronic inflammation and pain Multifactorial DDD progression	[6,24,57]

**Table 4:** Therapeutic possibilities in DDD.

Category	Therapy	Key features	Benefits and limitations	References
Conservative Management	Physical therapy	Strengthens core and paraspinal muscles Improves posture and flexibility	Reduces mechanical strain First-line therapy Requires adherence	[6,24]
	Lifestyle modifications (weight loss, smoking cessation, ergonomics)	Addresses modifiable risk factors	Slow degeneration Non-invasive Patient-dependent, needs adherence	[30,42,43]
	Pharmacological therapy (NSAIDs, muscle relaxants, analgesics)	NSAIDs ↓ COX activity and prostaglandins Analgesics reduce pain	Short-term relief; risks include gastrointestinal, renal, cardiovascular side effects	[86,121,122]
Medical pain Management	NSAIDs	Anti-inflammatory via COX inhibition	Short-term relief Limited disc penetration Systemic risks	[87,121]
	Opioids	Activate GPCR pathways → inhibit neurotransmission	Effective for severe pain High risk of addiction and dependence	[86,122]
	Muscle relaxants	Reduce muscle spasm	Symptomatic relief Sedation risk	[86,122,123]
	Epidural corticosteroid injections	Reduce inflammation via COX & arachidonic acid inhibition	Temporary relief Systemic steroid effects No long-term benefit	[86,123]
Emerging pharmacologic therapies	Pamidronate	Bisphosphonate inhibiting osteoclasts & bone turnover	Potential pain reduction Investigational	[26,57]
	Abaloparatide	Osteoporosis drug shown to reduce IVD degeneration (animal models)	Experimental No established human benefit	[22,107]
	Alpha-2-macroglobulin (A2M)	Protease inhibitor targeting FAC	Under investigation for slowing degeneration	[20,40]
Surgical management	Discectomy and decompression	Remove herniated disc tissue → relieve nerve compression	Effective for stenosis/herniation Do not halt degeneration	[52,84]
	Spinal fusion	Removes disc → inserts cage + hardware Eliminates motion	Pain reduction Decreases mobility Adjacent segment disease	[84,85,124]
	Total disc replacement	Replaces disc with motion-preserving prosthesis	Preserves mobility Modest benefits vs. fusion Long-term data limited	[7,30]
Biological and regenerative therapies	Platelet-rich plasma (PRP)	Growth factors (PDGF, VEGF, IGF-1, TGF-β) stimulate ECM synthesis	Promising early results Inconsistent human data No standardised protocols available	[86,125]
	Stem cell therapies	Implantation of regenerative cells into disc to restore ECM & hydration	Early promise Challenges: cell survival, microenvironment, delivery	[64,110,125]
	Growth factor therapy (TGF-β, BMP-7)	Enhances matrix production	Experimental Limited human evidence	[89,106,107,113]
	Gene therapy	Introduces genes to enhance matrix synthesis or reduce catabolism	Highly experimental Delivery and safety challenge	[89,107,113]

## Data Availability

The original contributions presented in this study are included in the article. Further inquiries can be directed to the corresponding author.
